# Pathogenic bacteria recovered from Gene X-pert tuberculosis-negative adult patients in Gondar, Northwest Ethiopia

**DOI:** 10.1186/s12890-023-02500-w

**Published:** 2023-06-06

**Authors:** Hana Yohannes, Teshome Belachew, Muluneh Assefa, Eden Getaneh, Haymanot Zeray, Asamirew Kegne, Samre Angawu, Gizeaddis Belay, Sirak Biset, Abiye Tigabu

**Affiliations:** 1 Department of Immunology and Molecular biology, School of Biomedical and Laboratory Sciences, College of Medicine and Health Sciences, University of Gondar, Gondar, Ethiopia; 2 Department of Medical Microbiology, School of Biomedical and Laboratory Sciences, College of Medicine and Health Sciences, University of Gondar, Gondar, Ethiopia; 3 University of Gondar Comprehensive Specialized Referral Hospital, University of Gondar, Gondar, Ethiopia

**Keywords:** Pathogenic bacteria, Lower respiratory tract infections, Gene X-pert

## Abstract

**Introduction:**

Lower respiratory tract infections (LRTIs) caused by drug-resistant pathogenic bacteria is a major problem in developing countries including Ethiopia. Therefore, this study aimed to determine the pathogenic bacteria and their antimicrobial susceptibility patterns among Gene X-pert tuberculosis-negative adult patients with clinically suspected LRTIs at the University of Gondar Comprehensive Specialized Referral Hospital, Gondar, Northwest Ethiopia.

**Methods:**

This institutional-based cross-sectional study was conducted from February 01 to March 15, 2020. Socio-demographic data were collected by using a structured questionnaire. A total of 254 sputum specimens were collected from Gene X-pert tuberculosis-negative patients. Bacterial recovery was performed using blood, chocolate, and MacConkey agar plates. Bacterial isolates were identified based on Gram staining, colony characteristics, and biochemical reactions. Antimicrobial susceptibility testing was performed using the Kirby-Bauer disk diffusion method. Methicillin resistance of *S. aureus* was confirmed using cefoxitin (30 µg). Descriptive statistics were calculated for each variable and results are shown in tables and figures.

**Results:**

In this study, the overall sputum culture positivity rate was 145/254 (57.1%). Gram-negative bacteria 111 (64.9%) were predominant compared to Gram-positive bacteria 60 (35.1%). Of the 145 culture-positive cases, 26 (14.8%) had poly-bacterial infections. *S. aureus* 40 (66.7%) was the predominant Gram-positive bacterium whereas *K. pneumoniae* 33 (29.7%), was the most isolated Gram-negative bacterium. Bacterial species, such as *S. aureus* were sensitive to ciprofloxacin 38/40 (95.0%), gentamicin 37/40 (92.5%), cefoxitin 36/40 (90.0%), and clindamycin 34/40 (85.0%). The proportion of Methicillin-resistant *S. aureus* was low, 4(10.0%). *S. pneumoniae* was sensitive to chloramphenicol 8/9 (88.9%) and resistant to ciprofloxacin 6/9 (66.7%). *K pneumoniae*, *P. aeruginosa, E. coli*, *Serratia* species, and *H. influenzae* also demonstrated high levels of resistance to ampicillin at rates of 21/33 (63.6%), 8/8 (100.0%), 15/17 (88.2%), 7/10 (70.0%), and 6/6 (100.0%), respectively.

**Conclusion:**

This study revealed a higher burden of Gram-negative and Gram-positive pathogenic bacterial agents, which is responsible for LRTs. Therefore, routine sputum culture identification and antibiotic susceptibility testing should be performed in Gene X-pert tuberculosis-negative patients.

## Introduction

Lower respiratory tract infections (LRTIs) are defined as infections that occur in the lower airways such as the trachea, bronchi, and lung tissue with clinical symptoms of cough, expectoration, dyspnea, wheezing, and/or chest pain usually for a period of 1 to 3 weeks [1]. It is a broad category of infections including acute bronchitis, bronchiectasis, bronchiolitis, emphysema, lung abscess, pleural effusion, chronic obstructive pulmonary disease, tuberculosis, and pneumonia [2]. Globally, LRTIs are a major threat to public health, causing significant morbidity and mortality in all age groups. The Global Burden of Disease Study in 2015 indicated that LRTIs were the leading infectious disease cause of death and the fifth leading cause of death overall [3]. LRTIs were the third leading cause of mortality in Brazil in 1990 and 2015, with 63.5 and 47.0 deaths/100,000 people, respectively. Although the number of deaths increased to 26.8%, mortality rates standardized by age were reduced by 25.5%, with an emphasis on children under 5 years of age [4]. In 2016 alone, LRTIs (defined as pneumonia, bronchitis, or bronchiolitis) caused an estimated 2.38 million deaths with a disproportionate effect on children younger than 5 years and adults more than 70 years old [5].

In African countries, LRTIs continue to be the leading cause of death due to the lack of identification of etiological agents and administration of appropriate medication. The burden and number of deaths among adults are higher in Sub-Saharan Africa than in other developed countries. For instance, 546.8 and 511.3 adult deaths per 100 000 were reported in Somalia and Chad, respectively; while the lowest reported mortality was in Finland in Western Europe, with 0.65 deaths per 100 000 [6, 7]. Aging, smoking, alcoholism, pulmonary disease, heart disease, immunosuppressive therapy, acute viral respiratory tract infection, cystic fibrosis, major surgical intervention, and malnutrition are among the major factors contributing to bacterial LRTIs [8].

Although the etiological agents of LRTIs differ in geographic location, the most common bacterial agents causing LRTIs are *S. pneumoniae*, *S. aureus, H. influenzae*, *K. pneumoniae, P. aeruginosa, E. coli*, and *A. baumannii* [9]. Respiratory tract epithelial damage due to viruses and transient immune suppression can cause *S. pyogenes* pneumonia [10]. In addition, hematogenous dissemination of intestinal bacteria to the lung and oropharyngeal aspiration causes LRTIs due to enteric bacteria [11]. Currently, inappropriate use and administration of antibiotics have resulted in treatment failure, increased hospital mortality rates, and healthcare-related expenditures for LRTI patients [12]. In Ethiopia, a few studies have been conducted on the etiology and antimicrobial susceptibility patterns of LRTIs in adult patients [13–18]. In the study area and most parts of the country, once patients suspected of tuberculosis had a negative Gene X-pert result, they were usually treated empirically or left without further infection diagnosis and medication. Therefore, this study investigated the pathogenic bacteria causing LRTIs among Gene X-pert tuberculosis-negative adult patients at the University of Gondar Specialized Referral Hospital, Northwest Ethiopia.

## Materials and methods

### Study design, period, and setting

This institutional-based cross-sectional study was conducted at the University of Gondar Comprehensive Specialized Referral Hospital, Gondar, Northwest Ethiopia from February 01 to March 15, 2020. Gondar town is located west of the northern Gondar administrative zone, which is 748 km from Addis Ababa, the capital city of Ethiopia [19]. The University of Gondar Comprehensive Specialized Referral Hospital is a teaching hospital that provides outpatient and inpatient services for more than five million people in North Gondar and its surrounding zones.

### Population, sample size, sampling technique, and data collection

Adult patients aged ≥ 18 years with clinical symptoms of LRTIs and those who consented to participate and provided sputum specimens were included in the study. However, patients who were on antimicrobial treatment and had a history of hospital admission 14 days before the data collection period were excluded from the study. A total of 254 LRTI-suspected adult patients with Gene X-pert tuberculosis negatives were enrolled. To determine the sample size, all patients who attended during the study period were selected using a convenient sampling technique. Sociodemographic information of the participants was collected using a pre-tested questionnaire. Gene X-pert negative LRTI was defined as patients with all TB signs and symptoms and Gene X-pert negatives. The Gene X-pert MTB/RIF assay procedure was performed at the University of Gondar Specialized and Referral Hospital tuberculosis laboratory and we have considered patients with negative Gene X-pert result for further identification of pathogenic bacteria.

### Sputum collection, processing, and bacterial identification

Purulent sputum specimens were collected in wide-mouthed sterile containers from each Gene X-pert pulmonary tuberculosis (PTB) negative patient. The quality of the collected sputum specimens was assessed using Bartlett’s scoring method by considering the score of pus cells, squamous epithelial cells, and macroscopic observation. A positive score (sum of positive and negative values assigned) on Gram’s stain was considered as an acceptable result for culture [20]. The specimens were then inoculated on chocolate agar, blood agar, and MacConkey agar plates (Oxoid Ltd., Basingstoke, UK). MacConkey agar plates were incubated aerobically at 37˚C for 24 h, whereas blood agar and chocolate agar plates were incubated in a humid atmosphere containing 5% carbon dioxide at 37 °C for 24 h. After 24 h of incubation, the plates were examined for bacterial growth, and preliminary identification was performed based on the Gram staining and colony characterization (size, shape, hemolysis pattern, and color). After obtaining pure colonies, further confirmatory identification was performed using standard microbiological techniques such as biochemical tests. A series of biochemical tests, including triple sugar iron agar, indole-motility, citrate, lysine decarboxylase, and urea were performed for Gram-negative bacterial isolates, whereas catalase, coagulase, bacitracin, and optochin disk tests were performed for Gram-positive bacteria [21, 22].

### Antimicrobial susceptibility testing

Antimicrobial susceptibility tests of the bacterial isolates were performed according to the Kirby-Bauer disk diffusion technique on Mueller-Hinton agar (Oxoid Ltd., Basingstoke, UK) with or without 5% lysed or non-lysed sheep blood [23]. About 2–3 colonies were picked with a wire loop and emulsified in 3 ml of sterile physiological saline. The turbidity of the bacterial suspension was matched and checked using the 0.5% McFarland standard. A sterile cotton swab was then dipped into the suspension and squeezed against the side of the test tube to avoid excess inocula. The test organisms were uniformly seeded on the surface of Mueller-Hinton agar using the lawn culture technique. After 5 min, a set of selected antimicrobial disks was aseptically placed on Mueller-Hinton agar plates and allowed to stand at room temperature for 15 min. Then, all plates were incubated for 24 h while maintaining all the requirements of the respective bacteria as done during the isolation process. The diameters of the zone of inhibition around the disk were measured using a ruler and compared with the Clinical Laboratory Standard Institute 2018 reference points. Results were interpreted as sensitive, intermediate, and resistant. An inhibition zone diameter of ≤ 21 mm was reported methicillin-resistant and ≥ 22 mm was considered methicillin-sensitive. The following routinely used antimicrobials were tested: ampicillin (10 µg), amoxicillin (10 µg), ciprofloxacin (5 µg), gentamicin (10 µg), ceftriaxone (30 µg), co-trimoxazole (25 µg), erythromycin (15 µg), clindamycin (30 µg), tetracycline (30 µg), chloramphenicol (10 µg), penicillin (10 µg), and cefoxitin (30 µg). All antibiotics were obtained from Abtek Biologicals, Ltd., Liverpool, UK [23].

### Quality control

Participant data were collected using a pre-tested questionnaire. All samples were collected and processed according to the standard operating procedure of specimen collection. The qualities of the specimens were checked based on Bartlett’s criteria [24]. The sterility of the culture media was ensured by incubating 5% of each batch of the prepared media at 37oc for 24 h. The performance of all media was checked by inoculating standard ATCC strains. To standardize the inoculum density of bacterial suspension, turbidity was adjusted using, 0.5 McFarland standard [23].

### Data analysis and interpretation

Data cleaning was done using EPI info version 7.1 and exported to SPSS version 20.0 for analysis. Descriptive statistics were then computed to calculate the frequencies. Data were summarized using numbers, percentages, graphs, and tables.

## Results

### Socio-demographic characteristics of the participants

A total of 254 LRTI-suspected patients were enrolled in this study. More than half, 149 (58.7%) of them were males and 138 (54.3%) were living in the urban. About 118 (46.5%) were in the age group of 18–35 years. One hundred fifteen (45.3%) of the study participants had 2–4 family members and 82 (32.2%) were unable to write. Moreover, 186 (73.2%) participants were married (Table 1).

**Table 1:** Socio-demographic characteristics of the study participants (N = 254)

Variables		Frequency (%)
Gender	Male	149 (58.7)
	Female	105 (41.3)
Residence	Urban	138 (54.3)
	Rural	116 (45.7)
Age in years	18–35	118 (46.5)
	36–55	91 (35.8)
	> 55	45 (17.7)
Family size	2–4 members	115 (45.3)
	5–7 members	103 (40.5)
	8 and above	36 (14.2)
Educational level	Unable to write	82 (32.2)
	Read and write	71 (28.0)
	Primary	32 (12.6)
	Secondary	33 (13.0)
	Degree and above	36 (14.2)
Marital status	Single	62 (24.4)
	Married	186 (73.2)
	Widowed	6 (2.4)

### The prevalence of pathogenic bacteria in LRTIs

**Fig. 1:**
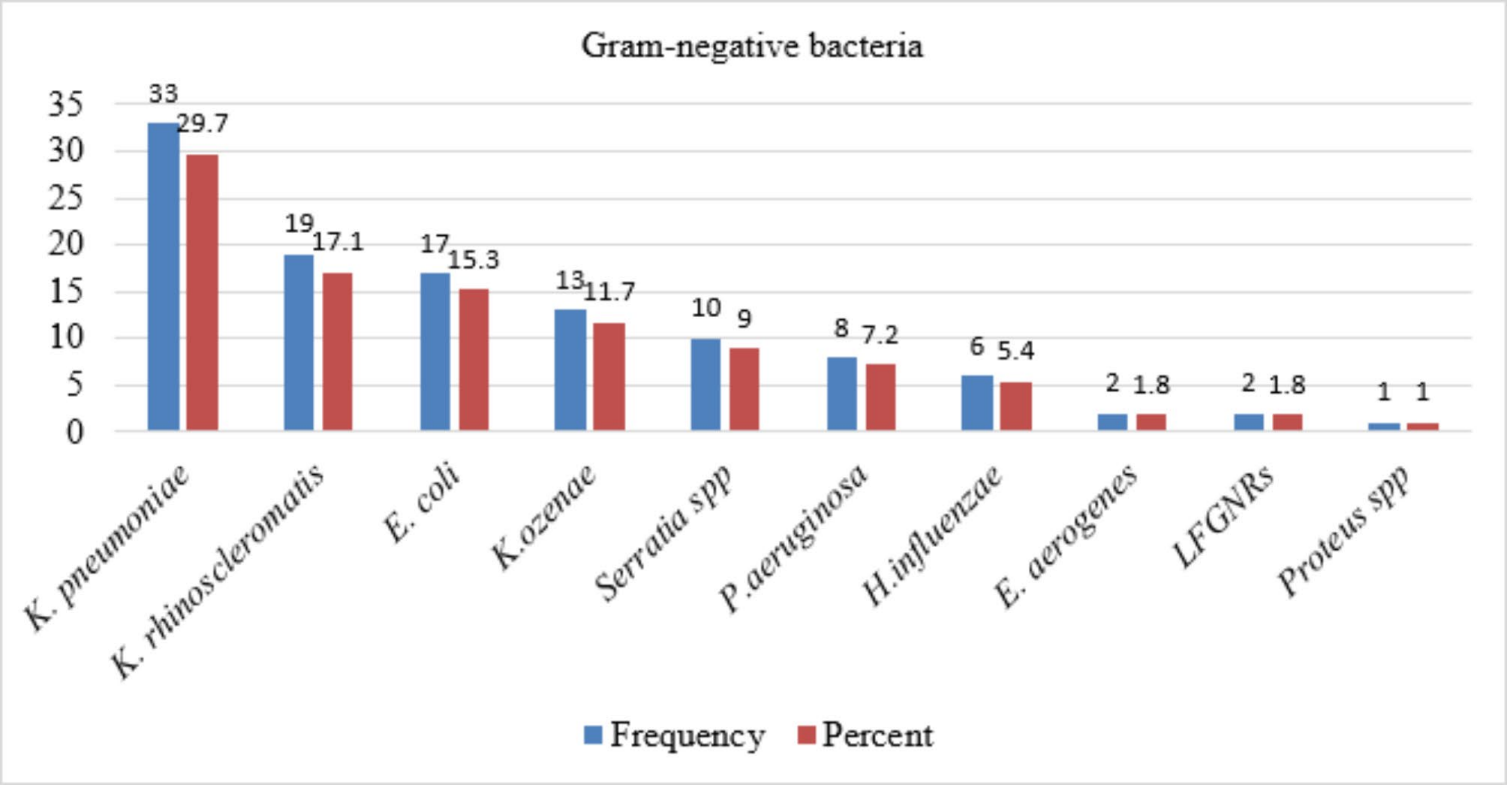
Frequency and percentage of Gram-negative bacteria from sputum specimen (N = 111)

In this study, the overall sputum culture-positivity rate among LRTI-suspected patients was 145/254 (57.1%). A total of 171 bacterial isolates were recovered, and about 26 (14.8%) patients were positive for more than one bacterial isolate. Gram-negative bacteria 111 (64.9%) were predominant over Gram-positive bacteria 60 (35.1%). Among Gram-negative bacterial isolates, *K. pneumoniae* 33 (29.7%) was the most frequently isolated bacteria, followed by *K. rhinoscleromatis* 19 (17.1%), *E. coli* 17 (15.3%), *K. ozienae*, 13 (11.7%), and *Serratia* species 10 (9.0%) (Fig. 1).

**Fig. 2:**
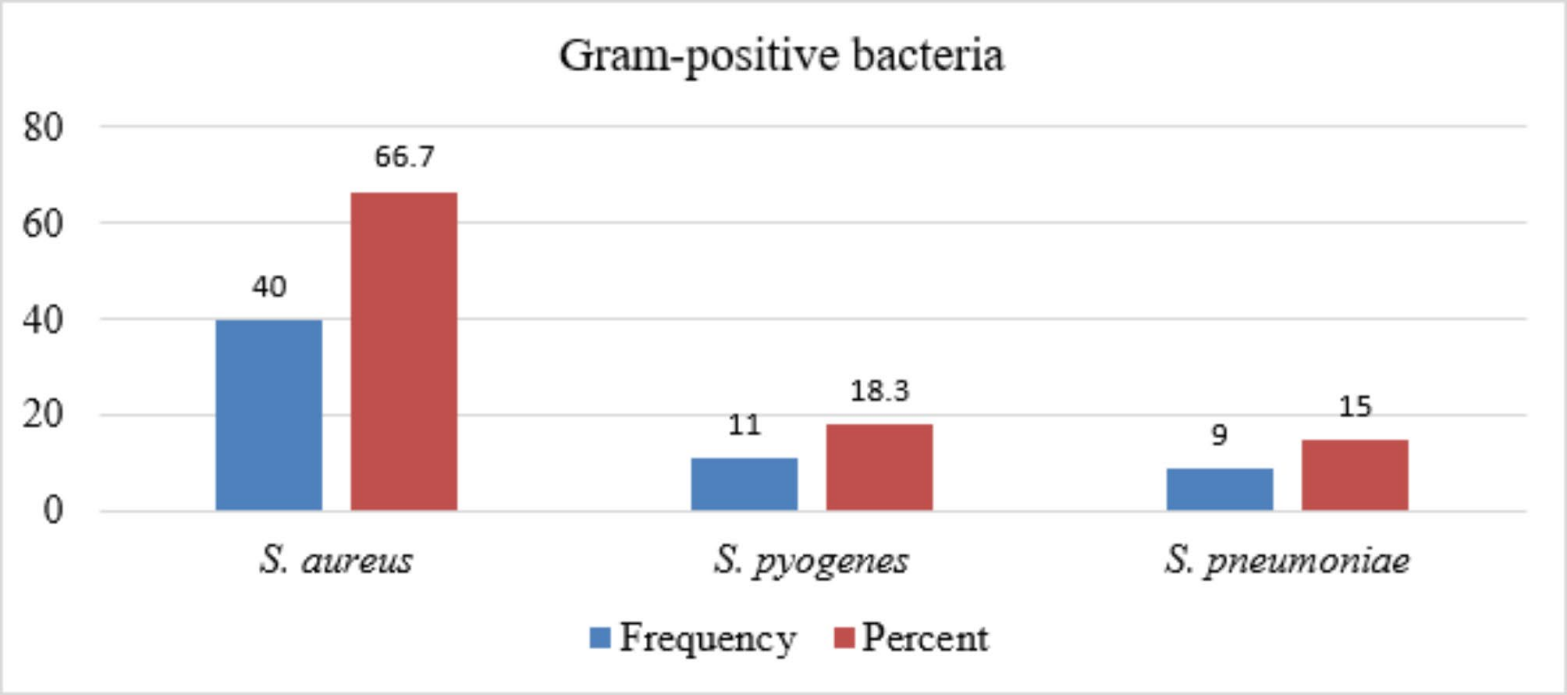
Frequency and percentage of Gram-positive bacteria from sputum specimen (N = 60)

Alternatively, *S. aureus*, *S. pyogenes*, and *S. pneumoniae* were among Gram-positive bacteria isolated with a frequency of 40 (66.7%), 11 (18.3%), and 9 (15.0%), respectively (Fig. 2).

### Antimicrobial susceptibility patterns of bacterial isolates

In this study, the antimicrobial susceptibility patterns of both Gram-positive and Gram-negative bacterial isolates were characterized (Tables 2 and 3). *S. aureus*, one of the leading Gram-positive bacteria isolated, demonstrated sensitivity to ciprofloxacin 38/40 (95.0%), gentamicin 37/40 (92.5%), cefoxitin 36/40 (90.0%), and clindamycin 34/40 (85.0%), but resistance to tetracycline, 22/40 (55.0%). *S. aureus* was resistant to methicillin 4/40 (10.0%). *S. pyogenes* and *S. pneumoniae* showed significant levels of resistance to some tested antimicrobial agents. For instance, *S. pyogenes* was resistant to ciprofloxacin, vancomycin, and erythromycin accounting for 8/11(72.7%) each. Similarly, *S. pneumoniae* showed significant levels of resistance to ciprofloxacin, erythromycin, and penicillin with the rate of 6/9 (66.7%), 6/9 (66.7%), and 7/9 (77.8%), respectively. However, chloramphenicol 11/11 (100.0%) and cefepime 9/9 (100.0%) showed good effects on *S. pyogenes* and *S. pneumoniae*, respectively (Table 2).

**Table 2:** Antimicrobial susceptibility profile of Gram-positive bacterial isolates (N = 60)

Antibiotics	Bacterial isolates					
	*S. aureus* (n = 40)		*S. pyogenes* (n = 11)		*S. pneumoniae* (n = 9)	
	S (%)	R (%)	S (%)	R (%)	S (%)	R (%)
**Gentamicin**	37 (92.5)	3 (7.5)	N/A	N/A	N/A	N/A
**Chloramphenicol**	N/A	N/A	11 (100.0)	0 (0)	8 (88.9)	1 (11.1)
**Ciprofloxacin**	38 (95.0)	2 (5.0)	3 (27.3)	8 (72.7)	3 (33.3)	6 (66.7)
**Ampicillin**	N/A	N/A	8 (72.7)	3 (27.3)	N/A	N/A
**Penicillin**	N/A	N/A	N/A	N/A	2 (22.2)	7 (77.8)
**Vancomycin**	N/A	N/A	3 (27.3)	8 (72.7))	6 (66.7)	3 (33.3)
**Clindamycin**	34 (85.0)	6 (15.0)	7 (63.6)	4 (36.4)	N/A	N/A
**Cefoxitin**	36 (90.0)	4 (10.0)	N/A	N/A	8 (88.9)	1 (11.1)
**Tetracycline**	18 (45.0)	22 (55.0)	N/A	N/A	N/A	N/A
**Erythromycin**	26 (65.0)	14 (35.0)	3 (27.3)	8 (72.7)	3 (33.3)	6 (66.7)
**Cefepime**	N/A	N/A	N/A	N/A	9 (100.0)	0 (0)

**Note.** S = Sensitive, R = Resistant, N/A = Not Applicable

Most *K. pneumoniae* demonstrated sensitivity to most of the tested antimicrobial agents. It showed 31/33 (93.9%) sensitive to gentamicin, 29/33 (87.9%) to chloramphenicol, 29/33 (87.9%) to ceftriaxone, 25/33 (75.8%) to cotrimoxazole, 30/33 (90.9%) to ceftazidime, and 26/33 (78.8%) to tetracycline. However, it was significantly ampicillin-resistant, 21/33 (63.6%). Likewise, bacterial isolates such as *P. aeruginosa, E. coli*, *Serratia* species, and *H. influenzae* demonstrated high levels of resistance to ampicillin at the rate of 8/8 (100.0%), 15/17 (88.2%), 7/10 (70.0%), and 6/6 (100.0%), respectively (Table 3).

**Table 3:** Antimicrobial susceptibility profile of Gram-negative bacterial isolates (N = 111)

Antibiotics	Bacterial isolates															
	*K. pneumoniae* (n = 33)		*P. aeruginosa* (n = 8)		*E. coli* (n = 17)		*Serratia spp* (n = 10)		*H. influenzae* (n = 6)		*E. aerogenes* (n = 2)		*Proteus spp* (n = 1)		LFGNRs (n = 2)	
	S (%)	R (%)	S (%)	R (%)	S (%)	R (%)	S (%)	R (%)	S (%)	R (%)	S (%)	R (%)	S (%)	R (%)	S (%)	R (%)
**GEN**	31 (93.9)	2 (6.1)	8 (100.0)	0 (0)	9 (52.9)	8 (47.1)	9 (90.0)	1 (10.0)	N/A	N/A	2 (100.0)	0 (0)	1 (100.0)	0 (0)	2 (100.0)	0 (0)
**CAF**	29 (87.9)	4 (12.1)	7 (87.5)	1 (12.5)	16 (94.1)	1 (5.9)	9 (90.0)	1 (10.0)	5 (83.3)	1 (16.7)	2 (100.0)	0 (0)	1 (100.0)	0 (0)	2 (100.0)	0 (0)
**CTR**	29 (87.9)	4 (12.1)	5 (62.5)	3 (37.5)	15 (88.2)	2 (11.8)	8 (80.0)	2 (20.0)	6 (100.0)	0(0)	2 (100.0)	0 (0)	1 (100.0)	0 (0)	2 (100.0)	0 (0)
**AMP**	12 (36.4)	21 (63.6)	0(0)	8 (100.0)	2 (11.8)	15 (88.2)	3 (30.0)	7 (70.0)	0 (0)	6 (100.0)	1 (50.0)	1 (50.0)	1 (100.0)	0 (0)	1 (50.0)	1 (50.0)
**COT**	25 (75.8)	8 (24.2)	5 (62.5)	3 (37.5)	14 (82.4)	3 (17.6)	6 (60.0)	4 (40.0)	N/A	N/A	1 (50.0)	1 (50.0)	1 (100.0)	0 (0)	2 (100.0)	0 (0)
**CAZ**	30 (90.9)	3 (9.1)	N/A	N/A	16 (94.1)	1 (5.9)	8 (80.0)	2 (20.0)	5 (83.3)	1 (16.7)	2 (100.0)	0 (0)	1 (100.0)	0 (0)	2 (100.0)	0 (0)
**TE**	26 (78.8)	7 (21.2)	6 (75.0)	2 (25.0)	15 (88.2)	2 (11.8)	8 (80)	2 (20)	N/A	N/A	2 (100)	0 (0)	1 (100)	0 (0)	2 (100)	0 (0)

**Note**: GEN: Gentamicin, CAF: Chloramphenicol, CTR: Ceftriaxone, AMP: Ampicillin, COT: Co-trimoxazole, CAZ: Ceftazidime, TE: Tetracycline, S: Sensitive, R: Resistant, N/A = Not Applicable, LFGNRS = Lactose fermenter Gram-Negative Rod, spp: species

## Discussion

In recent decades, antimicrobial resistance in the lower respiratory tract bacterial pathogens has become a major problem in developing countries, where the infectious disease burden is high that resulting in increased healthcare costs and mortality of patients [25, 26]. In this study, 145 (57.1%) respiratory cultures yielded growth for different bacteria from the 254 LRTI-suspected adult patients. Comparable findings were reported in India, 57.4% [27] and 52.8% [28]. This finding is higher than the previous reports from Ethiopia in Hawassa, 33.5% [18], Dessie, 38.7% [16], Bahr Dar, 40.3% [15], Arba Minch, 40.0% [13], Addis Ababa, 32.1% [17], Jimma, 45.0% [29], Mekelle, 43.7% [14], Cameroon, 46.8% [30], India, 17.0% [31]; 38.5% [32]; 43.3% [33], Nepal, 44.4% [34]; 39.7% [35], Nigeria, 14.5% [36];18.9% [37]; 24.2% [38]; 41.2% [39]; 46.1% [40], Tanzania, 20.4% [41], Sudan, 23.6% [42]; 42.0% [43], Central Kerala, 26,3% [2], and Sri Lanka, 29.4% [44]. However, our finding is lower than studies from Bangladesh, 64.0% [45], India, 65.1% [46]; 76.7% [47]; 83.0% [48]; 84.7% [49]; 86.1% [50], Ghana, 84.9% [51], and China, 70.9% [52]. This difference might be due to the variation in the bacterial distribution in geographic areas, study period, study population, the type and quality of the respiratory specimens used, and delay in specimen transportation.

This study demonstrated the predominance of pathogenic Gram-negative bacteria in LRTIs, accounting for 111 (64.9%), while 60 (35.1%) were Gram-positive bacteria. This is consistent with previous Ethiopian studies, which found a higher prevalence of Gram-negative bacteria, ranging from 56.7 to 77.8% than other respiratory pathogens [17, 29, 53]. Similar findings have also been reported from studies worldwide, 58.0% in Ghana [51], 70.9% [28] and 89.9% [33] in India,77.6% in Nepal [9], 82.5% in Nigeria [38], and 82.8% in China [54]. This is due to their strong pathogenic ability and virulence factors such as Type 1 pili may enhance the ability of gram-negative bacteria to adhere and colonize the lower respiratory tract, the unequal distribution of patients with community-acquired and hospital-acquired infections, and improper use of antibiotics results in increased drug-resistant clones.

In this study, about 26 (14.8%) sputum samples were positive for more than one bacterial isolate. Similarly, 7.4% had mixed infections in Arba Minch [13], 5.2% in Addis Ababa [17], 4.5% in Jimma [29], 13.3% in India [33], and 20.0% in Nepal [9]. The variation may relate to the sample size, geographic location, and use of empirical treatment. The identification of polymicrobial infection is crucial for treatment strategies to target clinically resistant strains through combination therapies.

In this study, *S. aureus* was found to be the most common Gram-positive bacteria recovered from sputum specimens of patients suspected of LRTIs, with a frequency of 40 (66.7%); consistent with studies in Pakistan reported by Khawaja et al. [55]. Unlikely, in several studies, *S. pneumoniae* was reported to be the foremost Gram-positive isolate causing pneumonia as reported in Ethiopia [15], Cameroon [56], Ghana [51], Pakistan [57], and China [52]; 60 (35.9%), 42 (38.9%), 24 (26.7%), 49 (30.8%), and 79 (32.6%), respectively. The primary reason for the increased prevalence of *S. aureus* is its widespread presence in the hospital and community settings. It is also a skin and mucous membrane flora that may invade the broken skin or adhere to medical equipment, spread to the lung through blood, and cause serious pulmonary infections in adult patients. Additionally, the ability of *S. aureus* to adapt to the milieu of the respiratory tract, its metabolic versatility, the ability to scavenge iron, coordinate gene expression, the horizontal acquisition of genes, and the expression of surface adhesins facilitates its persistence in the airways that increased its burden as a respiratory pathogen [58]. Among Gram-negative bacteria, *K. pneumoniae* was the most frequently isolated bacteria with a frequency of 33 (29.7%). This pathogen was also reported as the most important isolate by Ethiopian studies from Dessie [16] and Tigray [14], and elsewhere in Egypt [59], Nepal [60], and India [61].

*S. aureus*, the most frequently isolated bacteria, was sensitive to ciprofloxacin 38/40 (95.0%), gentamicin 37/40 (82.5%), cefoxitin 36/40 (90.0%), clindamycin 34/40 (85.0%), and erythromycin 26/40 (65.0%). Similarly, a study from Nigeria reported that the sensitivity of *S. aureus* to ciprofloxacin, gentamicin, and clindamycin was 100.0%, 100.0%, and 90.0%, respectively [36]. A study conducted in Jimma reported *S. aureus* resistance to ciprofloxacin (31.3%), gentamicin (31.5%), and erythromycin (75.0%) [29]. Nowadays, MRSA has become a significant problem in community and hospital settings, with high mortality rates [62]. Too in this study, the prevalence of MRSA was 4 (10.0%). Comparable findings were reported in Central Kerala, India, 15.4% [2], and 16.6% [28]. Higher MRSA was reported by Tewodros et al. [16], Cox, D. [63], and Koripella et al. [61]; 34.5%, 33.0%, and 26.7%, respectively.

In our study, of 33 *K. pneumoniae* isolates subjected to antimicrobial susceptibility testing; resistance rate was 2 (6.1%), 3 (9.1%), 4 (12.1%), 4 (12.1%), 7 (21.2%), and 8 (24.2%) to gentamicin, ceftazidime, chloramphenicol, ceftriaxone, tetracycline, and co-trimoxazole, respectively. Although *K. pneumoniae* become resistant to most antibiotics, our finding showed low resistance to certain antibiotics because of their minimal use as empirical therapy for adult LRTIs. Comparable results were reported by Temesgen et al. 6.7% and 20.0% for gentamicin and chloramphenicol [15]. Considerably, Kishimbo et al. reported resistance rates to ciprofloxacin, gentamicin, co-trimoxazole, and ceftriaxone; 17.4%, 26.1%, 43.5%, and 87.0%, respectively [41]. Similarly, 18.2% and 100% resistance to gentamicin and co-trimoxazole were reported by Regasa [64]. In contrast, a study conducted in Nigeria reported that the sensitivity of *K. pneumoniae* to gentamicin was 66.7%, respectively [36]. A study by Temesgen et al. [15] reported that *K. pneumoniae* isolates were found to be co-trimoxazole-resistant (90.0%), but sensitive to ceftriaxone (100.0%).

In the present study, *E. coli* showed 8 (47.0%) and 15 (88.0%) resistance to gentamicin and ampicillin, respectively. Correspondingly, 70.0% of *E. coli* showed resistance to ampicillin in Bahir Dar, Ethiopia [15]. A study from Addis Ababa also reported comparable *E. coli* resistance to gentamicin (40.0%) and ampicillin (80.0%) [17]. *P. aeruginosa* also isolates showed 3 (37.5%) resistance to both ciprofloxacin and co-trimoxazole, which correlates with a Tanzanian study that showed 37.5% resistance to ciprofloxacin and 37.0% to co-trimoxazole [41]. A study from Jimma also reported a 20.0% resistance of *P. aeruginosa* to ciprofloxacin [29]. Invariably, all *P. aeruginosa* isolates were piperacillin-resistant and this is in accordance with the results of a study conducted in Addis Ababa [17]. The difference in antibiotic-resistant bacterial strains and the proportion of identified bacteria in the study settings might be the reason for variation in the susceptibility pattern.

As strength, this study attempted to rule out PTB using Gene X-pert but did not consider the atypical bacteria, such as *M. pneumoniae, C. pneumoniae*, and *L. pneumophila*, due to their difficulty in growth using routine culture methods. Again, this study did not also consider anaerobic bacteria like; *Prevotella* spp., *Fusobacterium* spp., and *Clostridium* spp.) as routine culture methods are mostly aerobic. Hence, it can underestimate the actual prevalence of bacteria in the study area. Furthermore, this study did not attempt serotyping of *H. influenzae* and *S. pneumoniae* due to resource limitations.

## Conclusion

This study found a higher prevalence of pathogenic bacteria, 57.1% among PTB-negative adults in the study area. Both Gram-negative and Gram-positive bacterial agents were the frequently identified causes of LRTIs. *S. aureus* and *Klebsiella* species were the major bacterial pathogens responsible for LRTIs. *S. aureus* was highly susceptible to ciprofloxacin, gentamicin, cefoxitin, and clindamycin but resistant to tetracycline with the existence of MRSA strains. *S. pyogenes* and *S. pneumoniae* were highly ciprofloxacin-resistant. Gram-negative bacteria demonstrated a high level of susceptibility to chloramphenicol, ceftriaxone, and gentamicin but resistance to ampicillin. Therefore, confirmation of bacteria etiology after Gene X-pert detection of PTB is essential, and local drug susceptibility testing is a solution to drug resistance.

## Data Availability

All the necessary data are available within the manuscript.

## Abbreviations

MRSA: Methicillin-resistant *Staphylococcus aureus*
LRTI: Lower Respiratory Tract Infection
PTB: Pulmonary Tuberculosis

## References

[1] SInglA A, nKP MAMorIAVPJNJoLM (2021). Prevalence and Antimicrobial susceptibility pattern of bacterial agents involved in Lower respiratory tract infection at a Tertiary Care Hospital, Jaipur, Rajasthan, India. Natl J Laboartory Med.

[2] Regha I, Sulekha B (2018). Bacteriological profile and antibiotic susceptibility patterns of lower respiratory tract infections in a tertiary care hospital, Central Kerala. Int J Med Microbiol Trop Dis.

[3] Troeger C, Forouzanfar M, Rao PC, Khalil I, Brown A, Swartz S (2017). Estimates of the global, regional, and national morbidity, mortality, and aetiologies of lower respiratory tract infections in 195 countries: a systematic analysis for the global burden of Disease Study 2015. Lancet Infect Dis.

[4] Corrêa RdA, José BPdS, Malta DC, Passos VMdA, França EB, Teixeira RA (2017). Burden of disease by lower respiratory tract infections in Brazil, 1990 to 2015: estimates of the global burden of Disease 2015 study. Revista Brasileira de Epidemiologia.

[5] Collaborators GLRI (2018). Estimates of the global, regional, and national morbidity, mortality, and aetiologies of lower respiratory infections in 195 countries, 1990–2016: a systematic analysis for the global burden of Disease Study 2016. Lancet Infect Dis.

[6] Tchatchouang S, Bigna JJ, Nzouankeu A, Fonkoua M-C, Nansseu JR, Ndangang MS (2018). Prevalence of respiratory bacterial infections in people with lower respiratory tract infections in Africa: the BARIAFRICA systematic review and meta-analysis protocol. BMJ open.

[7] Amin B, Mohammed B, Kalgo Z (2019). Burden of Lower Respiratory Tract bacterial infection: a review. Bayero J Pure Appl Sci.

[8] Torres A, Peetermans WE, Viegi G, Blasi F (2013). Risk factors for community-acquired pneumonia in adults in Europe: a literature review. Thorax.

[9] Khan S, Priti S, Ankit S (2015). Bacteria etiological agents causing lower respiratory tract infections and their resistance patterns. Iran Biomed J.

[10] Akuzawa N, Kurabayashi M (2016). Bacterial pneumonia caused by streptococcus pyogenes infection: a case report and review of the literature. J Clin Med Res.

[11] Jonas M, Cunha BA (1982). Bacteremic Escherichia coli pneumonia. Arch Intern Med.

[12] Harris AM, Hicks LA, Qaseem A, Physicians HVCTFotACo C, ftCfD (2016). Prevention*. Appropriate antibiotic use for acute respiratory tract infection in adults: advice for high-value care from the American College of Physicians and the Centers for Disease Control and Prevention. Ann Intern Med.

[13] Regasa B (2014). Aetiology of bacterial pathogens from adult patients with community-acquired pneumonia in Arba Minch hospital, South Ethiopia. Science.

[14] Adhanom G, Gebreegziabiher D, Weldu Y, Gebreyesus Wasihun A, Araya T, Legese H et al. Species, risk factors, and antimicrobial susceptibility profiles of bacterial isolates from HIV-infected patients suspected to have pneumonia in Mekelle zone, Tigray, northern Ethiopia. BioMed research international. 2019;2019. 10.1155/2019/8768439 PMC6525850 31192259

[15] Temesgen D, Bereded F, Derbie A, Biadglegne F (2019). Bacteriology of community acquired pneumonia in adult patients at Felege Hiwot Referral Hospital, Northwest Ethiopia: a cross-sectional study. Antimicrob Resist Infect Control.

[16] Dessie T, Jemal M, Maru M, Tiruneh M. Multiresistant bacterial pathogens causing bacterial pneumonia and analyses of potential risk factors from Northeast Ethiopia. International Journal of Microbiology. 2021;2021. 10.1155/2021/6680343 PMC7964111 33763137

[17] Nurahmed N, Kedir S, Fantahun S, Getahun M, Mohammed A, Mohammed A (2020). Bacterial profile and antimicrobial susceptibility patterns of lower respiratory tract infection among patients attending selected health centers of Addis Ababa, Ethiopia. Egypt J Chest Dis Tuberculosis.

[18] Gebre AB, Begashaw TA, Ormago MD (2021). Bacterial profile and drug susceptibility among adult patients with community acquired lower respiratory tract infection at tertiary hospital, Southern Ethiopia. BMC Infect Dis.

[19] CSA E. Population projection of Ethiopia for all regions at wereda level from 2014–2017. Central Statistical Agency of Ethiopia; 2013.

[20] Goel R, Rangari AA, Tewari S. Analysis of Sputum Culture and Gram Staining in subjects with lower respiratory tract infection: a Microbiological Assessment. J Pharm Negat Results. 2022:262–5.

[21] Cusack T, Ashley E, Ling C, Rattanavong S, Roberts T, Turner P (2019). Impact of CLSI and EUCAST breakpoint discrepancies on reporting of antimicrobial susceptibility and AMR surveillance. Clin Microbiol Infect.

[22] M. C. District Laboratory Practice in Tropical Countries, Part 2. Cambridge, UK Cambridge University Press; 2006.

[23] Wayne P (2020). Clinical and laboratory standards institute. Performance standards for antimicrobial susceptibility testing. Inf SUPPL.

[24] Popova G, Boskovska K, Arnaudova-Danevska I, Smilevska-Spasova O, Jakovska T (2019). Sputum quality assessment regarding sputum culture for diagnosing lower respiratory tract infections in children. Open Access Macedonian Journal of Medical Sciences.

[25] Lawrence R. In: Jeyakumar E, editor. Antimicrobial resistance: a cause for global concern. Volume BMC proceedings. BioMed Central; 2013. 10.1186/1753-6561-7-S3-S1 PMC3892153 24268075

[26] Santella B, Serretiello E, De Filippis A, Folliero V, Iervolino D, Dell’Annunziata F (2021). Lower respiratory tract pathogens and their Antimicrobial susceptibility pattern: a 5-Year study. Antibiotics.

[27] Thomas AM, Jayaprakash C, Amma GMR (2016). The pattern of bacterial pathogens and their antibiotic susceptibility profile from lower respiratory tract specimens in a rural tertiary care centre. J Evol Med Dent Sci.

[28] Ramana K, Kalaskar A, Rao M, Rao SD (2013). Aetiology and antimicrobial susceptibility patterns of lower respiratory tract infections (LRTI’s) in a rural tertiary care teaching hospital in Karimnagar, South India. Am J Infect Dis Microbiol.

[29] Regasa B, Yilma D, Sewunet T, Beyene G (2015). Antimicrobial susceptibility pattern of bacterial isolates from community-acquired pneumonia patients in Jimma University specialized hospital, Jimma, Ethiopia. Saudi J Health Sci.

[30] Tchatchouang S, Nzouankeu A, Kenmoe S, Ngando L, Penlap V, Fonkoua M (2019). Bacterial aetiologies of lower respiratory tract infections among adults in Yaoundé, Cameroon. Biomed Res Int.

[31] Ahmed SM, Jakribettu RP, Meletath SK, Arya B, Shakir V (2013). Lower respiratory tract infections (LTRIs): an insight into the prevalence and the antibiogram of the gram negative, respiratory, bacterial agents. J Clin Diagn research: JCDR.

[32] Vijay S, Dalela G (2016). Prevalence of LRTI in patients presenting with productive cough and their antibiotic resistance pattern. J Clin Diagn research: JCDR.

[33] Singh S, Sharma A, Nag VL (2020). Bacterial pathogens from lower respiratory tract infections: a study from western Rajasthan. J Family Med Prim Care.

[34] Mishra S, Kattel H, Acharya J, Shah N, Shah A, Sherchand J (2012). Recent trend of bacterial aetiology of lower respiratory tract infection in a tertiary care centre of Nepal. Int J Infect Microbiol.

[35] Khushbu Y, Satyam P (2015). Bacteriological profile of lower respiratory tract infection (LRTI) among HIV seropositive cases in Central Terai of Nepal. Int J Curr Microbiol Appl Sci.

[36] Iregbu K (2018). Antibiogram of bacterial agents of lower respiratory tract infections in a central nigerian hospital. Niger J Med.

[37] Egbe CA, Ndiokwere C, Omoregie R (2011). Microbiology of lower respiratory tract infections in Benin City, Nigeria. Malaysian J Med sciences: MJMS.

[38] Akingbade O, Ogiogwa J, Okerentugba P, Innocent-Adiele H, Onoh C, Nwanze J (2012). Prevalence and antibiotic susceptibility pattern of bacterial agents involved in lower respiratory tract infections in Abeokuta, Ogun State, Nigeria. Rep Opin.

[39] Usman A, Amina M (2017). Isolation and identification of Bacteria Associated with Lower respiratory tract infection among patients attending General Hospital Katsina. J Microbiol Res.

[40] Olugbue V, Onuoha S. Prevalence and antibiotic sensitivity of bacterial agents involved in lower respiratory tract infections. Int J Biol Chem Sci. 2011;5(2).

[41] Kishimbo P, Sogone NM, Kalokola F, Mshana SE (2020). Prevalence of gram negative bacteria causing community acquired pneumonia among adults in Mwanza City, Tanzania. Pneumonia.

[42] Ahmed SMA-Z, Abdelrahman SS, Saad DM, Osman IS, Osman MG, Khalil EA (2018). Etiological trends and patterns of antimicrobial resistance in respiratory infections. Open Microbiol J.

[43] Ibrahim A. Bacterial etiology of community acquired pneumonia and their antimicrobial susceptibility in patients admitted to alshaab teaching hospital. Sudan Med Lab J. 2018;6(1).

[44] Amarasinghe N, Athavan M, Jayamanne D, Rajapakshe Y, Sadikeen A, Gunasekara K (2018). Bacterial profile and antibiotic susceptibility pattern of adult lower respiratory tract infections in Colombo, Sri Lanka. J Health Soc Sci.

[45] Ullah B, Ahmed S, Shahariar M, Yesmine S (2016). Current trend of antibiotic resistance in lower respiratory tract infections (LRTIs): an experience in a teaching hospital in Bangladesh. Bangladesh Pharm J.

[46] Elumalai A, Raj M, Abarna V, Bagyalakshmi R, Reddy S (2015). Study of Gram negative bacterial isolates from lower respiratory tract infections (LRTI) and their antibiogram pattern in a tertiary care hospital in south India. JMSC.

[47] Srivastava P, Kumar P, Nirwan P, Sharma M. Bacteriological profile and antibiogram pattern of lower respiratory tract infections in a tertiary care hospital in Northern India. Int J Pharm Res Bio-Science. 2013;2(3).

[48] Panda S, Nandini BP, Ramani T (2012). Lower respiratory tract infection-bacteriological profile and antibiogram pattern. Int J Curr Res Rev.

[49] Siddalingappa C, Kalpana L, Puli S, Vasudha T, Acharya A (2013). Sensitivity pattern of bacteria causing respiratory tract infections in a tertiary care centre. Int J Basic Clin Pharmacol.

[50] Bajpai T, Shrivastava G, Bhatambare G, Deshmukh A, Chitnis V (2013). Microbiological profile of lower respiratory tract infections in neurological intensive care unit of a tertiary care center from Central India. J basic Clin Pharm.

[51] Pappoe F, Amissah I (2014). Prevalence of bacterial pathogens isolated from sputum cultures of hospitalized adult patients with community-acquired pneumonia at the Cape Coast Teaching Hospital, Ghana. J Med Res.

[52] Tao L-L, Hu B-J, He L-X, Wei L, Xie H-m, Wang B-Q (2012). Etiology and antimicrobial resistance of community-acquired pneumonia in adult patients in China. Chin Med J.

[53] Dessie T, Jemal M, Maru M, Tiruneh M. Multiresistant bacterial pathogens causing bacterial pneumonia and analyses of potential risk factors from Northeast Ethiopia. Int J Microbiol. 2021:6680343. 10.1155/2021/6680343 PMC7964111 33763137

[54] Duan N, Du J, Huang C, Li H (2020). Microbial distribution and antibiotic susceptibility of lower respiratory tract infections patients from pediatric ward, adult respiratory ward, and respiratory intensive care unit. Front Microbiol.

[55] Khawaja A, Zubairi ABS, Durrani FK, Zafar A (2013). Etiology and outcome of severe community acquired pneumonia in immunocompetent adults. BMC Infect Dis.

[56] Ngekeng S, Pokam BT, Meriki HD, Njunda AL, Assob JCN, Ane-Anyangwe I. High prevalence of bacterial pathogens in sputum of tuberculosis suspected patients in Buea. Microbiol Res J Int. 2016:1–8.

[57] Ahmad I, Saleha S, Rahim K, Basit A, Rahman H, Qasim M (2017). Awareness of diverse bacterial flora distribution causing pneumonia in Dir.

[58] Parker D, Prince A, editors. Immunopathogenesis of Staphylococcus aureus pulmonary infection. Seminars in immunopathology. Springer; 2012. 10.1007/s00281-011-0291-7 PMC3577067 22037948

[59] Aboshanab K, Abdelaziz S, Hassouna NA, Aboulwafa MM (2015). Antimicrobial resistance pattern of some bacterial pathogens involved in lower respiratory tract infections in Egypt. Acta Microbiol.

[60] Ojha CR, Rijal N, Khagendra K, Palpasa K, Kansakar P, Gupta B (2015). Lower respiratory tract infections among HIV positive and control group in Nepal. VirusDisease.

[61] Koripella L, Perala BMK, Cheemala SS, Bhavani B (2016). Bacterial profile in sputum samples of pneumonia cases in a tertiary care hospital. Int J Res Rev.

[62] Vanamala K, Tatiparti K, Bhise K, Sau S, Scheetz MH, Rybak MJ (2021). Novel approaches for the treatment of methicillin-resistant Staphylococcus aureus: using nanoparticles to overcome multidrug resistance. Drug Discovery Today.

[63] Cox D. Antibiotic resistance: the race to stop the silent tsunami facing modern medicine. The Guardian. 2015.

[64] Regasa B. Drug resistance patterns of bacterial pathogens from adult patients with pneumonia in Arba Minch hospital, South Ethiopia. Glob J Med Res. 2014;14(5).

